# Altered Bacterial-Fungal Interkingdom Networks in the Guts of Ankylosing Spondylitis Patients

**DOI:** 10.1128/mSystems.00176-18

**Published:** 2019-03-26

**Authors:** Ming Li, Bingbing Dai, Yawei Tang, Lei Lei, Ningning Li, Chang Liu, Teng Ge, Lilong Zhang, Yao Xu, Yuqi Hu, Pengfei Li, Yan Zhang, Jieli Yuan, Xia Li

**Affiliations:** a College of Basic Medical Science, Dalian Medical University, Dalian, China; b Department of Rheumatology and Immunology, Dalian Municipal Central Hospital Affiliated of Dalian Medical University, Dalian, China; c Liaoning International Travel Health Care Center, Dalian, China; d Department of Rheumatology and Immunology, The Second Affiliated Hospital of Dalian Medical University, Dalian, China; University of California, Riverside

**Keywords:** ankylosing spondylitis, dysbiosis, interkingdom network, microbiota, mycobiota

## Abstract

Intestinal bacterial dysbiosis has been increasingly linked to ankylosing spondylitis (AS), which is a prototypic and best studied subtype of spondyloarthritis (SpA). Fungi and bacteria coexist in the human gut and interact with each other. Although they have been shown to contribute actively to health or disease, no studies have investigated whether the fungal microbiota in AS patients is perturbed. In this study, fecal samples from 22 AS patients, with clinical and radiographic assessments, and 16 healthy controls (HCs) were collected to systematically characterize the gut microbiota and mycobiota in AS patients by 16S rRNA gene- and ITS2-based DNA sequencing. Our results showed that the microbiota of AS patients was characterized by increased abundance of *Proteobacteria* and decreased *Bacteroidetes*, which was contributed by enrichment of *Escherichia-Shigella*, *Veillonella*, *Lachnospiraceae* NK4A136 group, and reduction of *Prevotella* strain 9, *Megamona*, and *Fusobacterium*. The gut mycobiota of AS patients was characterized by higher levels of *Ascomycota*, especially the class of *Dothideomycetes*, and decreased abundance of *Basidiomycota*, which was mainly contributed by the decease of *Agaricales*. Compared to HCs, decreased ITS2/16S biodiversity ratios and altered bacterial-fungal interkingdom networks were observed in AS patients. Compared with nonsteroidal anti-inflammatory drugs (NSAIDs), treating AS patients with biological agents induced obvious changes in the gut mycobiota, and this result was highly associated with disease activity indexes, including AS disease activity index (ASDAS) C-reactive protein (asCRP), erythrocyte sedimentation rate (ESR), and Bath AS disease activity index (BASDAI). In addition, altered mycobiota in AS patients was also found associated with the degree of radiographic damage.

**IMPORTANCE** The human gut is colonized by diverse fungi (mycobiota), and fungi have long been suspected in the pathogenesis of SpA. Our study unraveled a disease-specific interkingdom network alteration in AS, suggesting that fungi, or the interkingdom interactions between bacteria and fungi, may play an essential role in AS development. However, our study is limited by sample size, and in-depth mechanism studies and additional large-scale investigations characterizing the gut mycobiome in AS patients are needed to form a foundation for research into the relationship between mycobiota dysbiosis and AS development.

## Abstract (precis)

The human gut is colonized by diverse fungi (mycobiota), and fungi have long been suspected in the pathogenesis of SpA. Our study unraveled a disease-specific interkingdom network alteration in AS, suggesting that fungi, or the interkingdom interactions between bacteria and fungi, may play an essential role in AS development. However, our study is limited by sample size, and in-depth mechanism studies and additional large-scale investigations characterizing the gut mycobiome in AS patients are needed to form a foundation for research into the relationship between mycobiota dysbiosis and AS development.

## INTRODUCTION

Spondyloarthritis (SpA) is a group of several related but phenotypically distinct disorders: psoriatic arthritis (PsA), arthritis related to inflammatory bowel disease (IBD), reactive arthritis, a subgroup of juvenile idiopathic arthritis, and ankylosing spondylitis (AS) (1). The exact pathogenesis of SpA remains unknown (2); however, altered immune responses toward gut microbiota under the influence of genetic and environmental factors have been shown in autoimmune diseases related to SpA (3–6).

Among the related disorders, AS is the prototypic and best studied subtype of SpA. Up to 70% of AS patients have subclinical gut inflammation and 5 to 10% of these patients have more severe intestinal inflammation that progresses to clinically defined IBD (7). As intestinal dysbiosis has been increasingly linked to IBD in recent years (8–10), it is reasonable to speculate a close link between gut microbiota and AS development (3, 11). Previous studies have shown that AS patients and a transgenic rat model of AS had increased immunoglobulin G (IgG) or proinflammatory cytokines in response to bacterial products such as outer membrane protein and lipopolysaccharide (LPS) (12, 13). A study of 10 patients by 16S ribosomal DNA sequencing analysis has shown dysbiosis in terminal ileum biopsy specimens of AS patients (14). A recent quantitative metagenomic study, based on deep shotgun sequencing using gut microbial DNA from 211 Chinese individuals, also showed that alterations of the gut microbiome were associated with the development of AS (15). Alterations in the gut microbial genera, such as lower abundance of *Bacteroides* (16), increase of *Prevotella* (17), and *Lachnospiraceae* subgroups, etc. (18) in IBD were highly in accordance with the patterns that were observed in AS patients.

Besides bacterial dysbiosis, a distinct alteration of fungal microbiota (mycobiota) was also identified in fecal samples from IBD patients (19). Although constituting only a small part of the gut microbiome (20), the mycobiota has been shown to contribute actively to health or disease in a complex manner (21, 22). Actually, fungi have long been suspected in SpA. For example, the anti-Saccharomyces cerevisiae antibodies (ASCA) were found to be associated with intestinal inflammation in SpA (23). β-1,3-Glucan, a fungal product, had been shown to trigger SpA in BALB/c ZAP-70W163C mutant (SKG) mice (24), and this response was mediated by interleukin-23 (IL-23)-provoked local mucosal dysregulation and cytokines driving SpA syndrome (25). Dectin-1, the C-type lectin-like pattern recognition receptor of β-1,3-glucan, and the downstream gene caspase recruitment domain-containing protein 9 (CARD9) are the common candidates for genetic studies in AS, PsA, and Crohn’s disease, as polymorphisms of these genes were found highly associated with AS risk (26, 27). However, although many studies have reported fungal microbiota dysbiosis in IBD (19, 28–32), no research has investigated whether the fungal microbiota in AS patients is perturbed.

In this study, we characterized both microbial and fungal microbiota in fecal samples from AS patients using high-throughput sequencing and analyzed the correlation between bacterial and fungal microbiota. We also compared the gut microbiomes of AS patients receiving different therapeutic regimens or with different disease activity parameters, including AS disease activity index (ASDAS) C-reactive protein (asCRP), erythrocyte sedimentation rate (ESR), and Bath AS disease activity index (BASDAI). Our study represented the first systematic analysis of the microbiome in AS patients, and data from this study provided a rationale to support the role of mycobiota dysbiosis in AS pathogenesis.

## RESULTS

### Study participant characteristics.

We included a total of 71 individuals in the current analysis, composed of 30 healthy controls (HCs) and 41 individuals with AS (see Fig. S1 in the supplemental material). Of the individuals with AS, 19.51% (*n* = 8) were newly diagnosed as having AS and 80.49% (*n* = 33) were patients that had the disease for different lengths of time and were treated by biological agents (BLs) or nonsteroidal anti-inflammatory drug (NSAIDs). Nineteen patients were excluded from this study due to medical histories of other diseases, and/or use of antibiotics, probiotics, prebiotics, or synbiotics before the collection of fecal samples. The 22 AS patients included in this study were all males with an average age of 34.86 years. Fourteen HCs were excluded for age and gender matching. The remaining 16 HCs were all males with an average age of 34.35 years (not significantly different from the HC group; *P* > 0.05). As expected, the majority of these patients were HLA-B27 positive (>85%) and with axial involvement (>94%). The disease activity parameters, including asCRP, ESR, and BASDAI were summarized in Table 1. The radiographic assessments showed that 22.73%, 45.45%, and 31.82% of the patients have II, III, and IV levels of structural damage in their spine, respectively. During follow-up, nine of the patients were at some time exposed to NSAIDs, and eight were treated by Etanercept, a BL that treats autoimmune diseases by interfering with tumor necrosis factor (TNF) (a soluble inflammatory cytokine) by acting as a TNF inhibitor.

**TABLE 1:** Baseline demographic, clinical, and radiographic characteristics of the AS patients

Characteristic^*a*^	Value for AS patients (*n* = 22)
Age, yr [mean (range)]	34.86 (15–58)
Male, %	100
Disease duration, yr [mean (range)]	9.60 (0.17–40)
HLA-B27 positive, %	85.71
Disease activity parameter	
asCRP, mg/dl [mean (range/normal range)]	14.52 (0.1–67/≦10)
ESR, mm/h [mean (range/normal range)]	27.53 (2–67/≦15)
Axial involvement, %	94.44
BASDAI, mean (range)	5.05 (2.1–9.4)
Imaging classification	
I, %	0
II, %	22.73
III, %	45.45
IV, %	31.82
Medication use	
NSAIDs,^*b*^ % (treatment time, yr)	40.91 (6.75)
BLs,^*c*^ % (treatment time, yr)	36.36 (1.62)
Treatment naive, %	22.73

a HLA-B27, human leukocyte antigen (HLA) class I molecule B27; asCRP, AS disease activity index (ASDAS) C-reactive protein; ESR, erythrocyte sedimentation rate; BASDAI, Bath AS disease activity index.

b NSAIDs, nonsteroidal anti-inflammatory drugs. The NSAIDs used by AS patients enrolled in this study include Celebrex, Diclofenac, and Loxoprofen Sodium.

c BLs, biological agents. The BL used by AS patients enrolled in this study is Etanercept.

10.1128/mSystems.00176-18.1 FIG S1 Recruitment of participants and the process of sample collection. Download FIG S1, TIF file, 0.09 MB. Copyright © 2019 Li et al. 2019 Li et al. This content is distributed under the terms of the Creative Commons Attribution 4.0 International license.

### Altered bacterial microbiota in AS patients.

**FIG 1:**
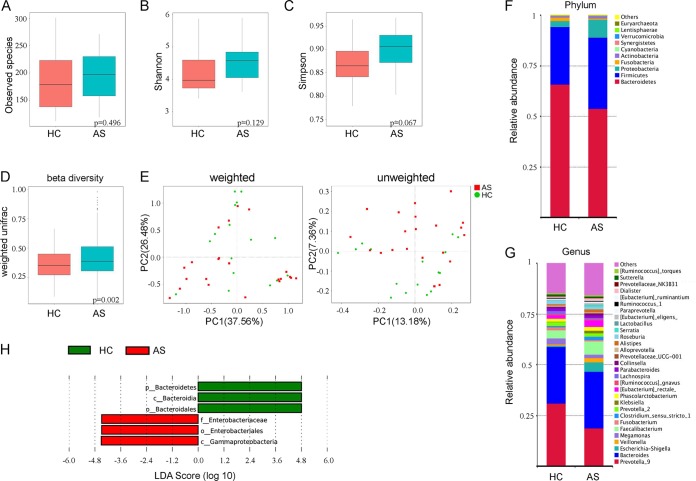
Altered bacterial microbiota biodiversity and composition in AS. (A to C) Observed species and Shannon and Simpson indexes describing the alpha diversity of the bacterial microbiota in two groups. (D) Beta diversity. (E) Principal-coordinate analysis (PCoA) of Bray-Curtis distance with each group colored according to the different treatment methods. PC1 and PC2 represent the top two principal coordinates that captured most of the diversity. The fraction of diversity captured by the coordinate is given as a percentage. Groups were compared by a Permanova method. (F and G) Global composition of bacterial microbiota at the phylum and genus levels. (H) Taxa differentiating AS from HC.

We first analyzed the bacterial fraction of the microbiota using high-throughput sequencing of the bacterial 16S rRNA gene. We obtained a total of 2,198,756 high-quality filtered reads from the fecal microbiota from 16 HCs (58,362 ± 5,810) and 22 AS patients (57,176 ± 4,879), with an average of 57,862 ± 5,400 reads aligned per sample. Compared with HCs, the observed species and alpha diversity (assessed using Shannon and Simpson indexes) of gut microbiota in AS patients was relatively increased, while there were no statistical differences among all indexes (Fig. 1A to C, all *P* > 0.05). The analysis based on weighted UniFrac showed a statistically significant increase in beta diversity in the AS group compared to the HC group (Fig. 1D, *P* = 0.0022), although the principal-coordinate analysis (PCoA) with weighted or unweighted UniFrac analysis did not exhibit an obvious separation between AS samples and HC samples (Fig. 1E).

The analysis of phylotypes indicated that *Bacteroidetes*, *Firmicutes*, *Proteobacteria*, *Fusobacteria*, and *Actinobacteria* were the dominant taxa in both the AS patients and healthy controls (Fig. 1F). At the phylum level, increased abundance of *Proteobacteria* (*P* = 0.0399) and decreased *Bacteroidetes* (*P* = 0.0177) were found in AS patients compared to the HCs (Fig. S2). We also observed a greater abundance of *Firmicutes* and *Actinobacteria* and a lower abundance of *Fusobacteria*, but these results were not statistically significant (all *P* > 0.05). At the genus level, enriched *Escherichia*-*Shigella* (*Proteobacteria*), *Veillonella* (*Firmicutes*), *Faecalibacterium* (*Firmicutes*), Eubacterium rectale group (*Firmicutes*), *Streptococcus* (*Firmicutes*), *Lachnospiraceae* NK4A136 group (*Firmicutes*), and reduced pattern of *Prevotella* strain 9 (*Bacteroidetes*), *Megamonas* (*Firmicutes*), and *Fusobacterium* (*Fusobacteria*) in AS patients were detected (Fig. 1G).

10.1128/mSystems.00176-18.2 FIG S2 Abundance of bacterial phyla in healthy control (HC) and ankylosing spondylitis (AS) groups. The *t* test was used to test the abundance of *Bacteroidetes* (0.6584 ± 0.1214 versus 0.5384 ± 0.1761; *P* = 0.01771) and *Proteobacteria* (0.0299 ± 0.0218 versus 0.0896 ± 0.1259; *P* = 0.0399). Download FIG S2, TIF file, 0.1 MB. Copyright © 2019 Li et al. 2019 Li et al. This content is distributed under the terms of the Creative Commons Attribution 4.0 International license.

A LefSe analysis was further adopted to identify the bacterial groups that showed significant differences in abundance between the AS and HC groups. As shown in Fig. 1H, the comparison between AS and HC groups revealed that the major depleted bacterial group in AS patients is the *Bacteroidetes* phylum, especially the *Bacterioidia* class and the *Bacteroidales* order. In contrast, *Enterobacteriales* and *Gammaproteobacteria* were significantly more abundant in the AS group (Fig. S3). The phylogenetic investigation of communities by reconstruction of unobserved states (PICRUSt) in the gut microbiota of AS patients and HCs showed that the gut bacteria in AS patients expressed more abundant genes involved in human diseases (especially infectious diseases), environmental information processing, and cellular processes (Fig. S4).

10.1128/mSystems.00176-18.3 FIG S3 The linear discriminant analysis (LDA) tree showing significantly different bacterial groups between HC and AS groups. o, order; c, class; f, family. Download FIG S3, TIF file, 0.2 MB. Copyright © 2019 Li et al. 2019 Li et al. This content is distributed under the terms of the Creative Commons Attribution 4.0 International license.

10.1128/mSystems.00176-18.4 FIG S4 Phylogenetic Investigation of Communities by Reconstruction of Unobserved States (PICRUSt) in the gut microbiota of AS patients and HCs. Download FIG S4, TIF file, 0.2 MB. Copyright © 2019 Li et al. 2019 Li et al. This content is distributed under the terms of the Creative Commons Attribution 4.0 International license.

### Altered bacterial microbiota in AS patients receiving different therapeutic regimens.

**FIG 2:**
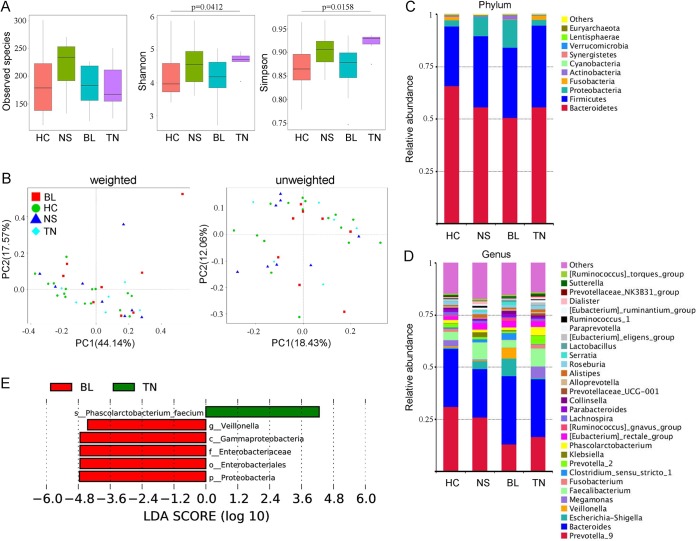
Altered bacterial microbiota biodiversity and composition in AS patients receiving different therapeutic regimens. (A) Observed species and Shannon and Simpson indexes describing the alpha diversity of the bacterial microbiota in the different groups. (B) Beta diversity. Principal-coordinate analysis (PCoA) of Bray-Curtis distance with each group colored according to the different treatment methods. PC1 and PC2 represent the top two principal coordinates that captured most of the diversity. The fraction of diversity captured by the coordinate is given as a percentage. Groups were compared by a Permanova method. (C and D) Global composition of bacterial microbiota at the phylum and genus levels. (E) Taxa differentiating the AS-BL group from the AS-TN group.

In our study, some of the fecal samples were from newly diagnosed AS patients without any medical treatment, and they were defined as the treatment-naive group (TN group) (*n* = 5). The other patients were grouped by the therapeutic regimens received, including biologics (BL) (*n* = 8) and NSAID (NS) (*n* = 9) (details in Table 1). Compared with healthy individuals, the species of gut bacteria in AS patients treated with NSAID were obviously increased, but the statistical test did not show significant differences between any two groups (Fig. 2A, all > 0.05). The Shannon and Simpson indexes suggested a significant elevation of alpha diversity in treatment-naive patients (*P* = 0.0412, *P* = 0.0158) compared to HCs. Among the AS patients, treatment with biologics resulted in reduction of alpha diversity of gut bacteria in contrast to the TN group, especially when tested by Simpson index (*P* = 0.0497). The principal-coordinate analysis (PCoA) by both weighted and unweighted UniFrac analysis showed that there was no obvious separation of groups (Fig. 2B). The variations of gut microbes in each group were also observed on phylum and genus levels (Fig. 2C and D). The *Proteobacteria* (especially the Enterobacteriaceae family) and the *Veillonella* genus were found enriched in AS patients treated with biologics compared with the patients without treatment (TN group), in which the species of Phascolarctobacterium faecium was significantly more abundant (Fig. 2E). However, no biomarkers were detected in others groups of AS patients by means of LefSe analysis.

### Altered mycobiota in AS patients.

**FIG 3:**
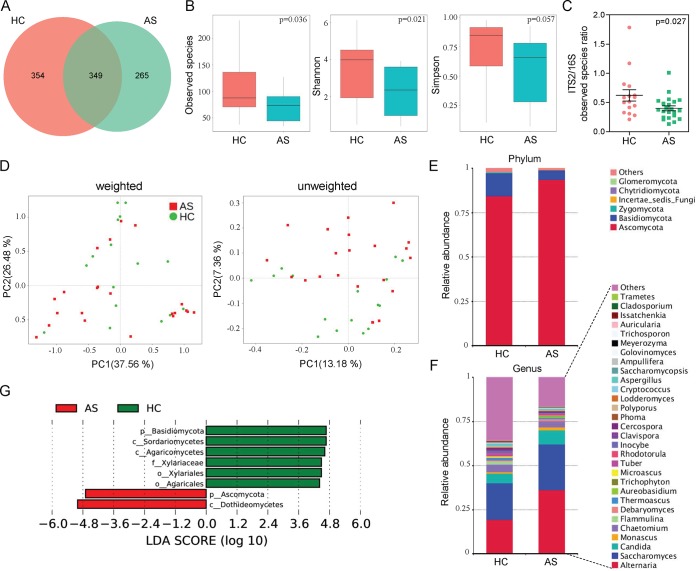
Altered fungal microbiota biodiversity and composition in AS. (A) The Venn diagram depicts OTUs that were unique to HC, unique to AS, or shared. (B) Observed species and Shannon and Simpson indexes describing the alpha diversity of the fungal microbiota in two groups. (C) ITS2/16S observed species ratio. (D) Beta diversity. PCoA of Bray-Curtis distance with each sample colored according to the treatment group (two groups). PC1 and PC2 represent the top two principal coordinates that captured most of the diversity. The fraction of diversity captured by the coordinate is given as a percentage. Groups were compared by a Permanova method. (E and F) Global composition of fungal microbiota at the phylum and genus levels. (G) Taxa differentiating AS from HC samples.

By sequencing ITS2, we obtained a total of 2,201,454 high-quality filtered reads from the fecal mycobiota of 16 HCs (57,247 ± 4,733) and 22 AS patients (58,463 ± 5,871), with an average 57,933 ± 5,372 reads aligned per sample. The results showed that there were 354 OTUs unique to the HC group and 265 OTUs unique to the AS group, while 349 OTUs were shared by the two groups (Fig. 3A). In contrast to results seen in the bacterial microbiota, the alpha diversity of intestinal fungi was significantly decreased in AS patients; as shown in Fig. 3B, the observed species and Shannon index in the AS group were significantly lower than in the control group (all *P* < 0.05). To explore the equilibrium between bacterial and fungal diversity in the gut, we determined the fungal-to-bacterial species ratio. This ratio was significantly decreased in AS samples (*P* = 0.027 [Fig. 3C]). The PCoA showed that AS samples grouped separately from HC samples, which indicated that changes in the fungal communities might be one of the factors influencing the disease (Fig. 3D and Fig. S5). A detailed comparison of the relative abundance of fungi in the HC and AS groups (Fig. 3E) showed that the *Ascomycota* and *Basidiomycota* phyla dominated in both groups and that there was an obvious change in the proportions of these two phyla in AS patients. Among the most dominant genera, *Alternaria*, *Saccharomyces*, and *Candida* were increased in AS patients, in contrast to decreases in other genera (Fig. 3F). The comparison between AS patients and HCs by LefSe revealed that the higher taxonomic levels of *Ascomycota*, especially the class of *Dothideomycetes* in this phylum, were significantly more abundant in AS patients (Fig. 3G), except for the family *Xylariaceae* (which belongs to *Ascomycota*), while the phylum *Basidiomycota* was dominant in HCs, which may primarily due to the abundance of *Agaricales*.

10.1128/mSystems.00176-18.5 FIG S5 PCoA of gut mycobiota in AS patients and HCs with center of mass. Download FIG S5, TIF file, 2.9 MB. Copyright © 2019 Li et al. 2019 Li et al. This content is distributed under the terms of the Creative Commons Attribution 4.0 International license.

### Altered mycobiota in AS patients receiving different therapeutic regimens.

**FIG 4:**
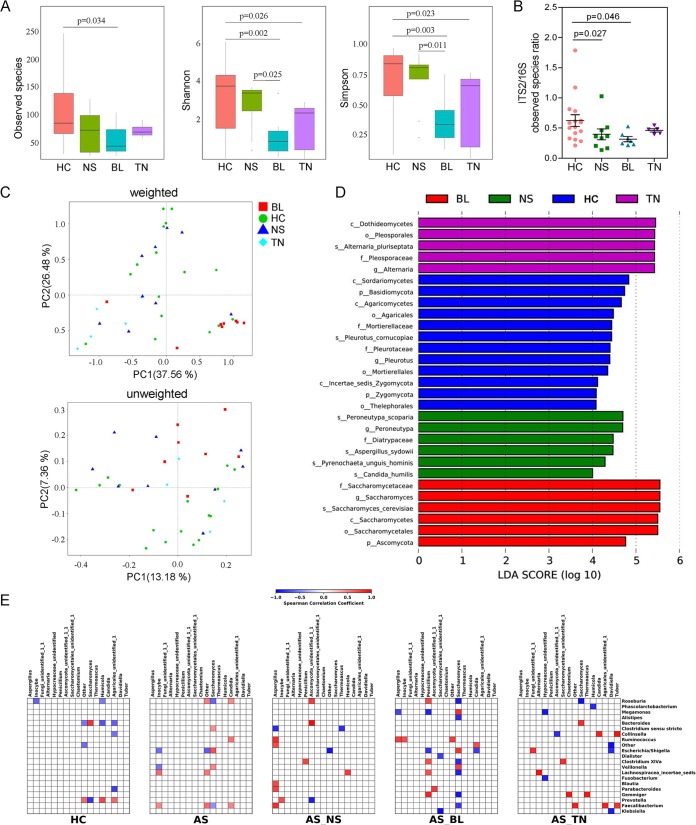
Altered mycobiota and bacterial-fungal correlation in AS patients receiving different therapeutic regimens. (A) Observed species, Shannon index, and Simpson index describing the alpha diversity of the fungal microbiota in four groups studied. (B) ITS2/16S observed species ratio. (C) Beta diversity. PCoA of Bray-Curtis distance with each sample colored according to the treatment group (four groups). PC1 and PC2 represent the top two principal coordinates that captured most of the diversity. The fraction of diversity captured by the coordinate is given as a percentage. Groups were compared by a Permanova method. (D) Main composition of fungal microbiota in four groups studied. (E) Specific bacterial-fungal correlation pattern in AS. Distance correlation plots of the relative abundance of fungal and bacterial genera. Statistical significance was determined for all pairwise comparisons; only significant correlations (*P* value of <0.05 after false discovery rate correction) are displayed. Positive values (blue squares) indicate positive correlations, and negative values (red squares) indicate inverse correlations. The shading of the square indicates the magnitude of the association; darker shades are more strongly associated than lighter shades. The sign (positive or negative) of the correlation was determined using Spearman’s method.

We further compared the gut mycobiota of AS patients grouped by different therapeutic regimens. Notably, a significantly reduced alpha diversity was observed in treatment-naive AS patients compared with the healthy controls, especially when evaluated by Shannon and Simpson indexes (Fig. 4A, all *P* < 0.05; see also Tables S1 to S3 in the supplemental material). Treatment with the biologic, Etanercept, resulted in an even lower level of observed fungal species and alpha diversity compared with the untreated TN group. In contrast, the NSAID treatment did not induce a distinct change in the number of gut fungal species in AS patients. However, when we evaluated diversity by using the fungal-to-bacterial diversity ratio, we observed a significant decreasing pattern in AS patients treated with both NSAID (Fig. 4B, *P* = 0.027) and Etanercept (*P* = 0.046), compared with that of the HC group. The PCoA by both weighted and unweighted Unifrac analysis showed that the gut mycobiota of the BL group separately clearly from the HC, NS, and TN groups, indicating that the treatment of Etanercept had a profound influence on the fungal communities in AS patients (Fig. 4C). The LefSe analysis revealed that the most dominant fungal microbiota differed significantly among the four groups (Fig. 4D). Notably, the fungal microbiota in treatment-naive AS patients was characterized by the dominance of the *Dothideomycetes* class, which was consistent with the results of Fig. 3. In the BL group, the most dominant fungal microbiota was *Saccharomyces*, and this genus contributed significantly to the abundance of *Ascomycota* in the AS patients of BL group.

10.1128/mSystems.00176-18.8 TABLE S1 Statistical test of the observed species for different groups. Download Table S1, DOCX file, 0.01 MB. Copyright © 2019 Li et al. 2019 Li et al. This content is distributed under the terms of the Creative Commons Attribution 4.0 International license.

10.1128/mSystems.00176-18.9 TABLE S2 Statistical test of the Shannon index values for different groups. Download Table S2, DOCX file, 0.01 MB. Copyright © 2019 Li et al. 2019 Li et al. This content is distributed under the terms of the Creative Commons Attribution 4.0 International license.

10.1128/mSystems.00176-18.10 TABLE S3 Statistical test of the Simpson index values for different groups. Download Table S3, DOCX file, 0.01 MB. Copyright © 2019 Li et al. 2019 Li et al. This content is distributed under the terms of the Creative Commons Attribution 4.0 International license.

### AS patients showed altered bacterial-fungal associations.

In addition to composition differences, we found that the bacterial and fungal microbiota network at the genus level in AS patients was notably different from that in healthy controls (Fig. S6). Specifically, the density of the bacterial network in AS patients was remarkably higher than that of the healthy individuals, while reduced network centralization and density of fungal communities were detected in these patients, which suggested an alteration of the entire ecosystem in the guts of AS patients. To test this hypothesis, we further investigated the bacterial-fungal correlation at the genus level according to disease phenotype. A higher Spearman correlation in AS patients compared with HCs was found (Fig. 4E). Interestingly, in AS patients, we observed a positive correlation between the abundance of *Saccharomyces* and *Clostridium sensu stricto*, *Escherichia*/*Shigella*, and *Veillonella* and a negative correlation between the abundance of *Saccharomyces*, *Roseburia*, and *Faecalibacterium*. A positive correlation between the abundance of *Candida* and *Roseburia*, *Faecalibacterium*, and *Ruminococcus* was also detected in AS patients, which differed from that of the HCs. Strikingly, treatment with Etanercept and NSAID induced extensive changes in bacterial-fungal associations in AS patients compared with the untreated AS patients. Notably, many positive correlations connecting genera from *Aspergillus* to *Ruminococcus*, *Blautia*, *Parabacteroides*, and *Faecalibacterium* were observed in AS patients treated with NSAID. There were more positive correlations between the abundance of *Penicillium* and *Clostridium* XIVa, *Roseburia*, *Lachnospiracea incertea sedis*, and *Gemmiger* in AS patients treated with Etanercept. Additionally, *Saccharomyces* followed a complicated inverse correlation with several bacterial genera in the BL group. Taken together, these results suggested a complex relationship between the bacteria and fungi in the gut microbiota and that specific alterations were present in patients receiving different therapeutic regimens.

10.1128/mSystems.00176-18.6 FIG S6 Bacterial and fungal microbiota network at the genus level in AS patients and HCs. Download FIG S6, TIF file, 1.1 MB. Copyright © 2019 Li et al. 2019 Li et al. This content is distributed under the terms of the Creative Commons Attribution 4.0 International license.

### Altered mycobiota in AS patients was associated with disease activity and degree of radiographic damage.

**FIG 5:**
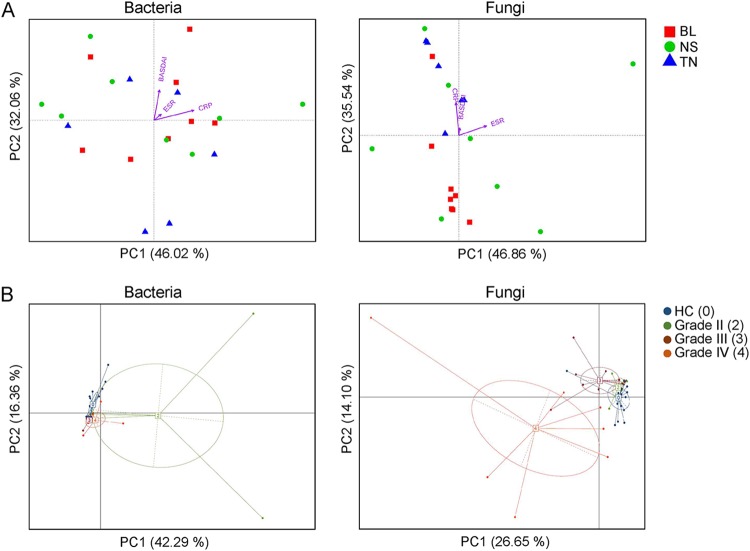
Altered mycobiota in AS patients is associated with disease activities and levels of radiographic damage. (A) The canonical correspondence analysis (CCA) establishes the relationship between the disease activity measures and the bacterial and fungal community in AS patients. The direction of the arrows indicates correlation with the first two canonical axes, and the length of the arrows represents the strength of the correlations. (B) PCA of the gut bacterial and fungal genera of AS patients according to their stages of radiographic changes.

Canonical correspondence analysis (CCA) was used to establish the relationship between AS disease activity indexes (including BASDAI, asCRP, and ESR) and the bacterial and fungal genera. As shown in Fig. 5A, the BASDAI and asCRP levels were found strongly correlated to the fungal genera in treatment-naive AS patients (TN), whereas no obvious correlations were detected between bacterial genera and the disease activity indexes. We further analyzed the gut bacterial and fungal compositions in AS patients at the genus level according to their stages of radiographic changes by principal-component analysis (PCA) (Fig. 5B). Intriguingly, a strongly separated pattern of gut mycobiota was observed in AS patients with grade III and grade IV stages, compared with the healthy controls and the grade II stage of AS patients. The elevated relative abundance of genera, such as *Saccharomyces*, in AS patients at grade IV stage may contribute to the alteration of fungal community patterns (Fig. S7).

10.1128/mSystems.00176-18.7 FIG S7 Relative abundance of fungal genera in AS patients with different degrees of radiographic damage. 2, 3, 4, imaging classification of radiographic damage. 0, the HCs without radiographic damage. Download FIG S7, TIF file, 0.1 MB. Copyright © 2019 Li et al. 2019 Li et al. This content is distributed under the terms of the Creative Commons Attribution 4.0 International license.

## DISCUSSION

In this study, we explored a distinct mycobiota pattern and altered bacterial-fungal interactions in the guts of AS patients, which represented a novel research viewpoint of the gut microbiome dysbiosis in AS.

Our finding provided a further confirmation of the alterations in gut microbial groups that might be associated with the development of AS. At the phylum level, 2.99-fold-increased abundance of *Proteobacteria* and 0.18-fold-decreased *Bacteroidetes* abundance were found in AS patients, which was shown in a previous study of the terminal ileum biopsy specimens of AS patients (14). Notably, the decrease of *Bacteroidales* in AS patients was mainly caused by the 39.53% decrease in *Prevotella* spp. This result apparently disagreed with the study of Wen et al. (15), in which an increase in the abundance of *Prevotella* spp. was observed in AS patients, although the study by Costello et al. supported that the level of the family *Prevotellaceae* was decreased in AS patients (14). *Prevotella* spp. were found to have proinflammatory properties by augmented release of inflammatory mediators from immune cells and various stromal cells (33), which suggested that some *Prevotella* strains might be clinically important pathobionts and could participate in human diseases by promoting chronic inflammation. However, on the other hand, a depletion of immune-stimulating *Prevotella* in the gut may be closely associated with immunodeficiency in humans, as supported by the fact that *Prevotella* abundance was found reduced in the guts of patients with AIDS (34) and within the lung microbiota in patients with asthma and chronic obstructive pulmonary disease (35).

Prompted by recent studies of IBD patients (20, 36), we profiled the fungal microbiome of the AS patients by sequencing analysis of the ITS2 marker gene, which provided greater resolution of the mycobiome membership compared to metagenomic and 18S rRNA gene sequencing data (20). Interestingly, a more pronounced fungal dysbiosis than bacterial dysbiosis in AS patients was detected in this study. We observed a significant decrease in the diversity of intestinal fungi in these patients. What is more, the abundance of *Ascomycota* and *Basidiomycota* were strongly negatively correlated with each other and were among the most important discriminative features between AS and HC mycobiota. These results highly agreed with findings in IBD patients, in which the *Basidiomycota*-to-*Ascomycota* abundance ratio differed between patients with IBD and HCs (19), which suggests that this imbalance may be either driven by inflammation or involved in the inflammatory process.

Fungi and bacteria coexist in the human and animal gut and interact with each other (37–39). Expansion or reduction of fungi was observed in mice after antibiotic treatment or after cessation of antibiotic treatment (40), suggesting a balance between fungal and bacterial microbiota. Our observation of the alterations in the fungal-bacterial diversity balance in AS suggested a modified interkingdom interaction. In addition to differences in the ITS2/16S biodiversity ratio, we noted a disease-specific pattern for the interkingdom network by the Spearman correlation analysis. In AS patients, especially the treatment-naive patients, the number and intensity of the correlations between fungi and bacteria were increased. The altered biodiversity in bacteria and fungi was associated with new interkingdom interactions that may be involved in the inflammatory process (19). Notably, this interaction in AS patients receiving NSAID or BL differed significantly from that of the HC and TN groups. Especially in patients treated with BL (Etanercept), the stronger correlations between fungi and bacteria suggested a profound effect of immunosuppressive regimens. While it is possible that time could affect the composition of the microbiome in AS patients, given the short length of Etanercept treatment in our study cohort (1.62 years) and the differences of gut microbiota between AS patients treated by NS and BL, the impact of medications used should not be neglected. Given the limited number of study cases, further large-scale studies on the characterization of gut microbiome and mycobiome in AS patients with different therapeutic regimens are necessary.

CRP is well established as a biomarker that directly reflects inflammation as an acute-phase reactant in AS (41). AS patients showed significant correlation between CRP with clinical parameters such as pain, morning stiffness, enthesitis-related local discomfort, BASDAI, BASFI (Bath ankylosing spondylitis functional index), and BASMI (Bath ankylosing spondylitis metrology index). In our study, we found a strong positive correlation between serum CRP levels and fungal microbiota in the new cases of AS (TN group), and this pattern was confirmed by the CCA of BASDAI. In contrast, treatment with BL or NSAID has profound effects on changing specific gut microbial and fungal groups, which may be associated with altered disease activities in AS patients. In addition, it was confirmed that disease activity contributed longitudinally to radiographic progression in the spines of AS patients (42). The structural damage in the spine was found to be associated with the acute-phase reactants (APR) CRP and ESR (43–46). We therefore analyzed the gut microbial and fungal microbiota structures according to the radiographs that was scored according to the New York criteria. Interestingly, the gut fungal microbiota of AS patients clearly clustered into three groups, and it was highly correlated with the radiographic assessment. The patients with level III and IV damage in their spines had different fungal microbiota structure than patients with level II damage or healthy controls, while no significant clustering was observed between the latter two groups. These results suggested a possible role of the mycobiome in the development of AS.

### Conclusion.

In conclusion, our study discovered a mycobiota dysbiosis in AS patients in addition to the alterations in bacterial microbiota. Moreover, we unraveled disease-specific interkingdom network alterations in AS that suggested that fungi, or the interkingdom interactions between bacteria and fungi, may be involved in the development of AS. Finally, although our study was not statistically sufficient, we identified some trends suggesting that different therapeutic regimens, especially biologics, may induce changes in both bacterial and fungal microbiota in AS. However, in the absence of a controlled diet and the low number of study participants, it should be mentioned that any variation observed could theoretically be the result of a diet bias. Further studies involving larger cohorts and metagenomics, or metabolomics, may elucidate the impact of bacterial and fungal microbiota on AS development.

## MATERIALS AND METHODS

### Study subjects and sample collection.

The recruitment of participants and the process of sample collection were depicted in Fig. S1 in the supplemental material. Forty-one patients (aged 15 to 58 years) were ultimately recruited from Dalian Municipal Central Hospital and the Second Affiliated Hospital of Dalian Medical University, Dalian, China, from May to September 2017. The disease activity measures of AS patients included the Bath AS disease activity index (BASDAI), AS disease activity index (ASDAS) C-reactive protein (asCRP), CRP, erythrocyte sedimentation rate (ESR), patient’s global assessment and spinal pain (42). Two readers independently scored the radiographs according to the New York criteria, which describe five grades of sacroiliitis ranging from 0 to 4 (47).

The fecal samples were collected in stool collection tubes (Stratec, Germany), which were prefilled with stool DNA stabilizer (Stratec, Germany) for collection, then frozen, and stored at −80°C for further use. All subjects were examined clinically before sampling and were subsequently divided into four groups according to different pharmacological therapies: treatment naive (TN) (*n* = 8), patients receiving nonsteroidal anti-inflammatory drug (NSAID) (*n* = 18), and patients receiving biologics (BL) (*n* = 15). The samples of the healthy controls (HCs) (*n* = 30) were collected during routine physical examination at the Liaoning International Travel Health Care Center, Dalian, China.

Individuals with the following diseases were excluded from the study: cardiovascular disease, diabetes mellitus, cirrhosis, infections with known active bacteria, fungi, or virus. Individuals who abused drug or alcohol in the last year or used antibiotics, probiotics, prebiotics, or synbiotics in the month before collection of the fecal samples were also excluded. There were no specific diet restrictions (vegan, vegetarian, etc.) for the study participants.

### DNA isolation and library construction.

The metagenomic DNA in 0.2 g of each fecal sample was extracted by the QIAamp DNA stool minikit (Qiagen, Germany). The purity and concentration of the metagenomic DNA were measured by using a NanoDrop 2000 spectrophotometer (Thermo Fisher Scientific, USA).

The V3-V4 region of 16S rRNA genes (representing bacteria) and the internal transcribed spacer region 2 (ITS2) (representing fungi) (19) were amplified with the primers (for 16S rRNA genes, primers F341 and R806 [PCR product, 425 bp]; for ITS2, primers ITS3 and ITS4 [PCR product, 320 bp]). Primer sets were modified with Illumina adapter regions for sequencing on the Illumina GAIIx platform, and reverse primers were modified with an 8-bp Hamming error-correcting barcode to differentiate samples. The DNA template (100 ng) was combined with 5 μl PCR buffer, 1 μl dNTPs, 0.25 μl HotStarTaq Plus DNA polymerase (Qiagen), and 2.5 pmol of each primer in a total volume of 50 μl. PCR consisted of an initial step at 95°C for 5 min; 25 (16S rRNA genes) or 38 (ITS2 rDNA) cycles, with 1 cycle consisting of 94°C for 45 s, 55°C for 45 s, and 72°C for 60 s; and a final extension at 72°C for 10 min. DNA products were checked by 1.5% (wt/vol) agarose gel electrophoresis in 0.5 mg/ml ethidium bromide and purified with the Qiaquick gel extraction kit (Qiagen).

### Bioinformatic analysis.

Sequences of the V3-V4 region of 16S rRNA genes and ITS2 were detected using an Illumina HiSeq PE250 platform (reconstructed cDNA sequence: 2 × 250 bp, Novogene Bioinformatics Technology Co. Ltd., Beijing, China). The Ribosomal Database Project (RDP) Classifier 2.8 was used for taxonomical assignment of all sequences at 50% confidence after the raw sequences were identified by their unique barcodes. OTUs present in 50% or more of the fecal samples were identified as core OTUs. The observed species and Shannon and Simpson indexes were calculated with QIIME (version 1.9.1). The abundance and diversity of the OTUs (beta diversity) were examined using principal-coordinate analysis (PCoA) with weighted or unweighted UniFrac analysis in R software. The linear discriminant analysis (LDA) effect size analysis (LEfSe) was used with the Kruskal-Wallis rank sum test to detect features with significantly different abundances between assigned taxa, and the linear discriminant analysis was performed to estimate the effect size of each feature. The bacterial groups with LDA score of ≧4.00 were shown as significantly abundant group in the indicated group. Canonical correlation analysis (CCA) was used to establish the relationship between environmental factors (including CRP, ESR, and BASDAI) and the bacterial community in fecal samples from AS patients as described previously (48). The correlations between gut bacterial and fungal genera were determined using Spearman’s method as described previously (19).

### Statistical analysis.

All data were evaluated as means ± SEM. Statistical analysis of the quantitative multiple group comparisons was performed using one-way analysis of variance (and nonparametric), followed by Wilcox’s test; when two groups were compared, the nonparametric *t* test was performed with the assistance of GraphPad Prism 6 (Graph Pad Software, La Jolla, CA, USA). Results were considered to be statistically significant with *P* < 0.05.

### Ethics statement.

This study protocol was approved by the Ethics Committees of all participating hospitals, including Dalian Municipal Central Hospital and the Second Affiliated Hospital of Dalian Medical University, Dalian, China. All the procedures were performed in accordance with the guidelines approved by the Ethics Committee of Dalian Medical University, China. After receiving a written description of the aim of this study, all participants gave written informed consent prior to enrollment.

### Data availability.

The sequence data were deposited in NCBI Sequence Read Archive (SRA) with accession numbers PRJNA525366 and PRJNA525614.
