# Supplementary figures and images for: Altered Bacterial-Fungal Interkingdom Networks in the Guts of Ankylosing Spondylitis Patients
Source: mSystems. 2019 Mar 26;4(2):e00176-18. doi: 10.1128/mSystems.00176-18 (PMC6435815; doi:10.1128/mSystems.00176-18)

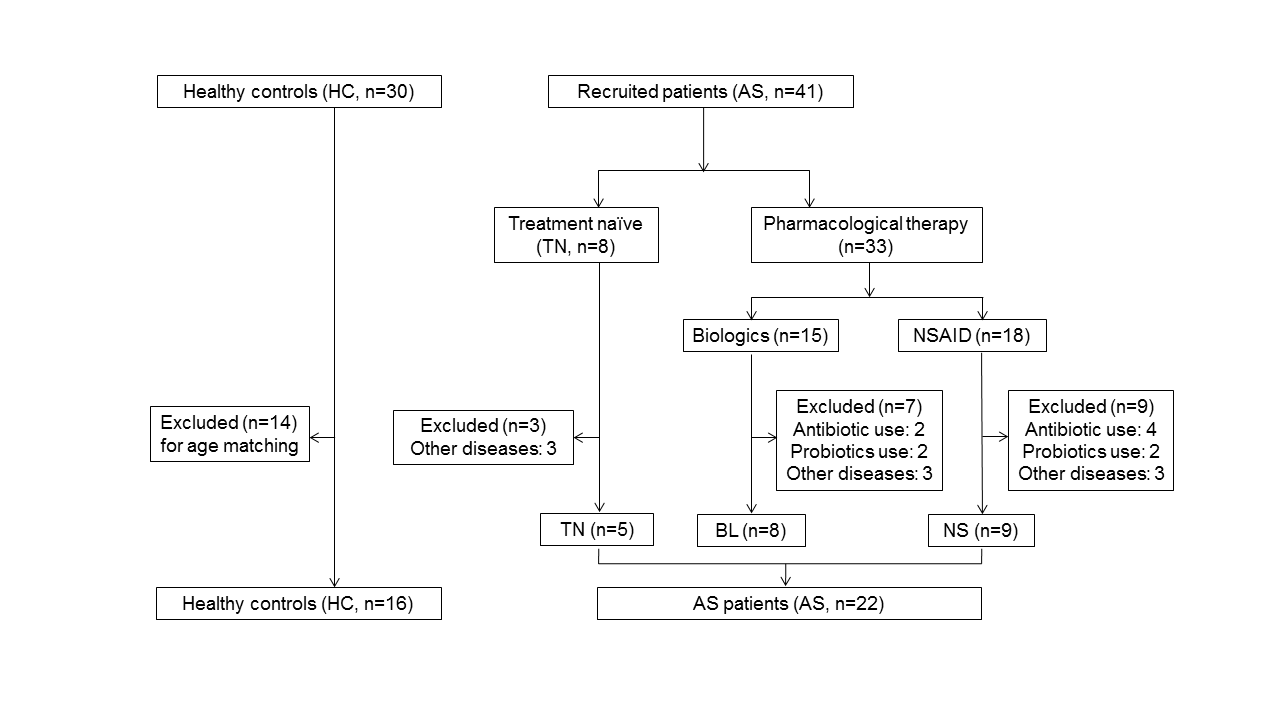

Supplement: FIG S1 [file mSystems.00176-18-sf001.tif]

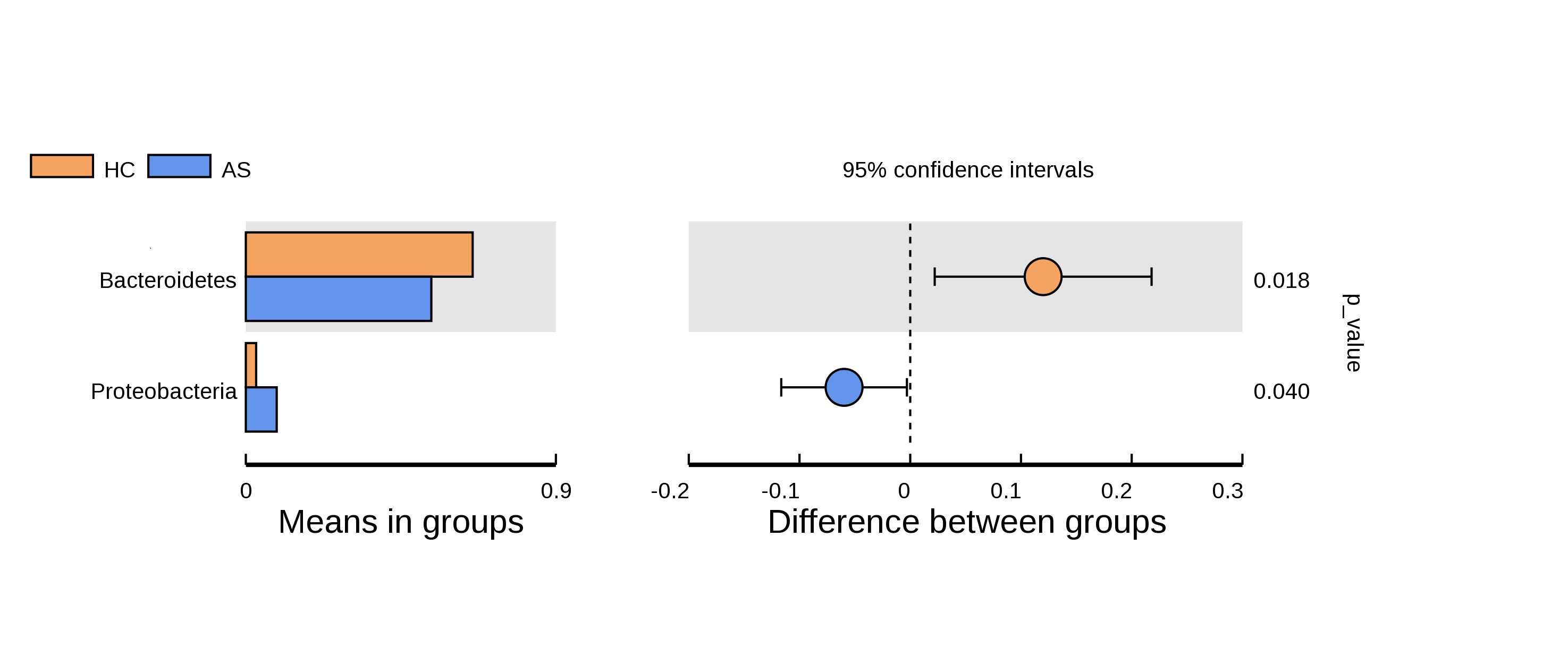

Supplement: FIG S2 [file mSystems.00176-18-sf002.tif]

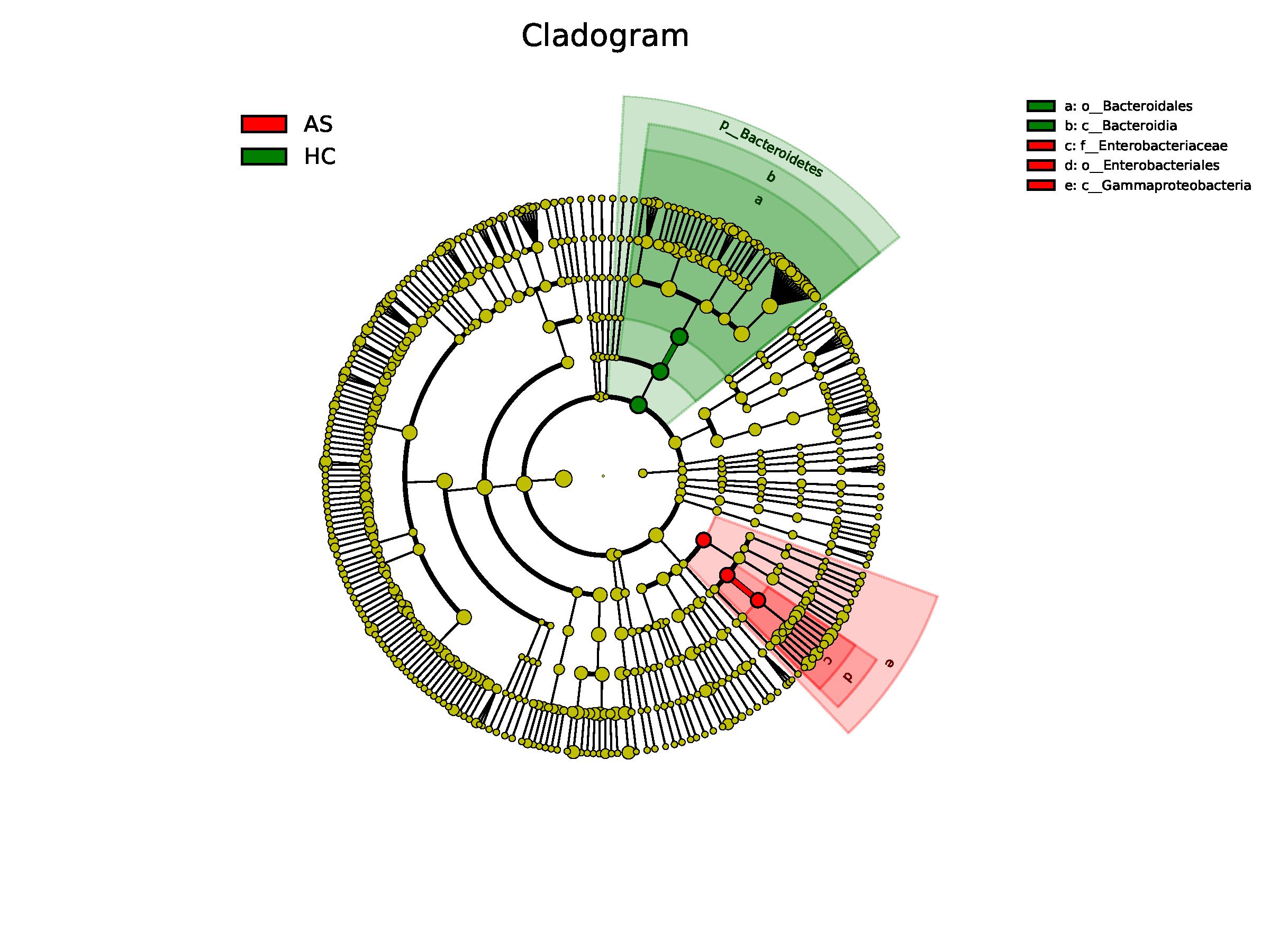

Supplement: FIG S3 [file mSystems.00176-18-sf003.tif]

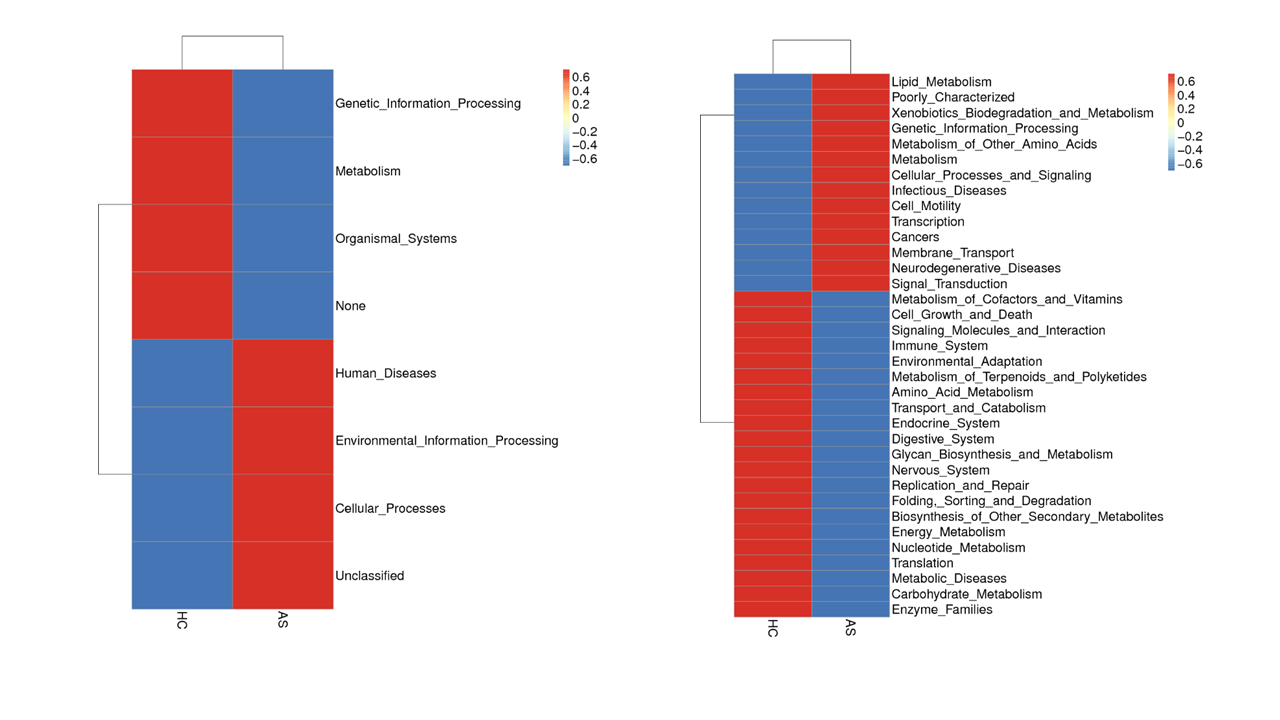

Supplement: FIG S4 [file mSystems.00176-18-sf004.tif]

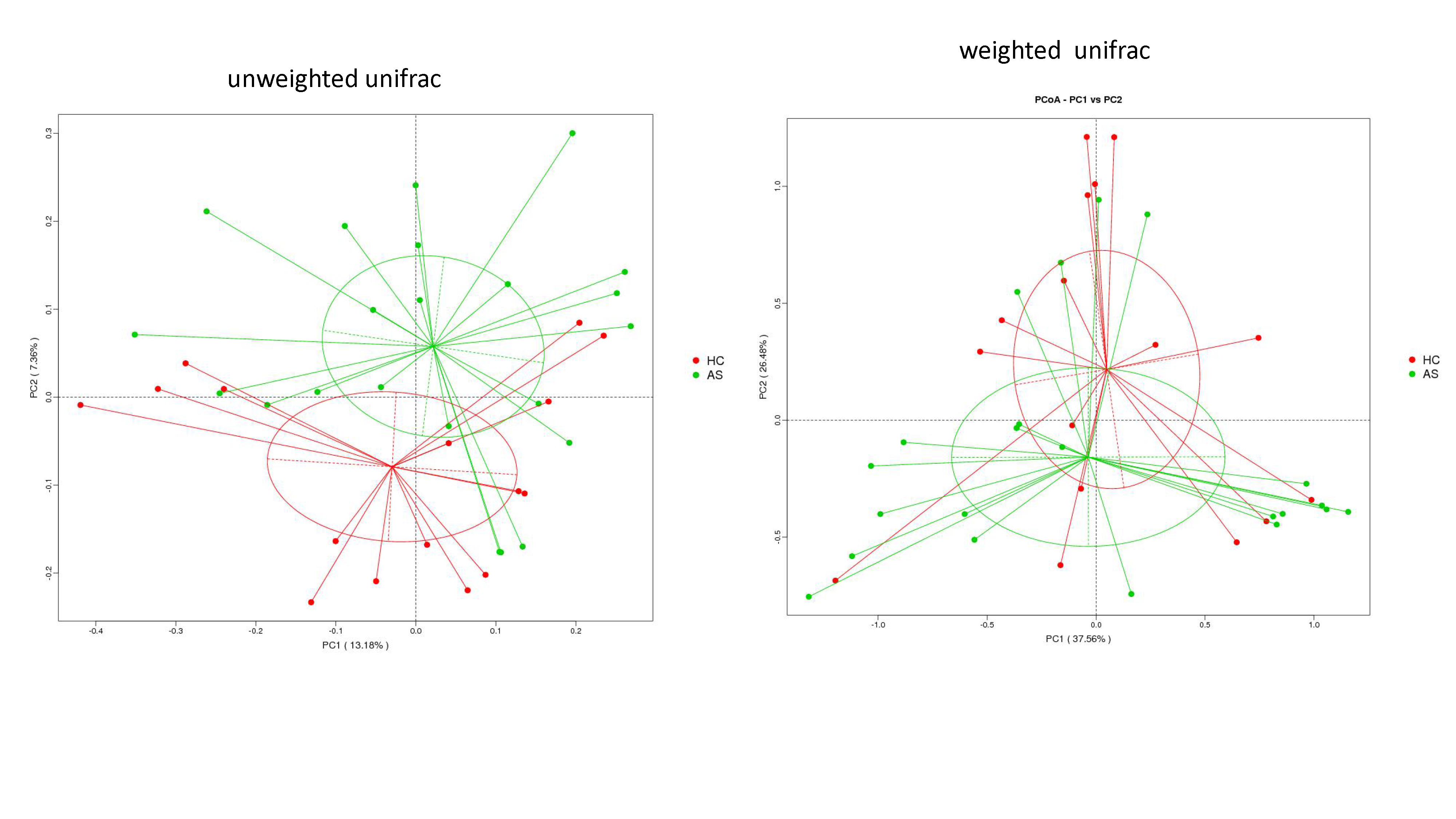

Supplement: FIG S5 [file mSystems.00176-18-sf005.tif]

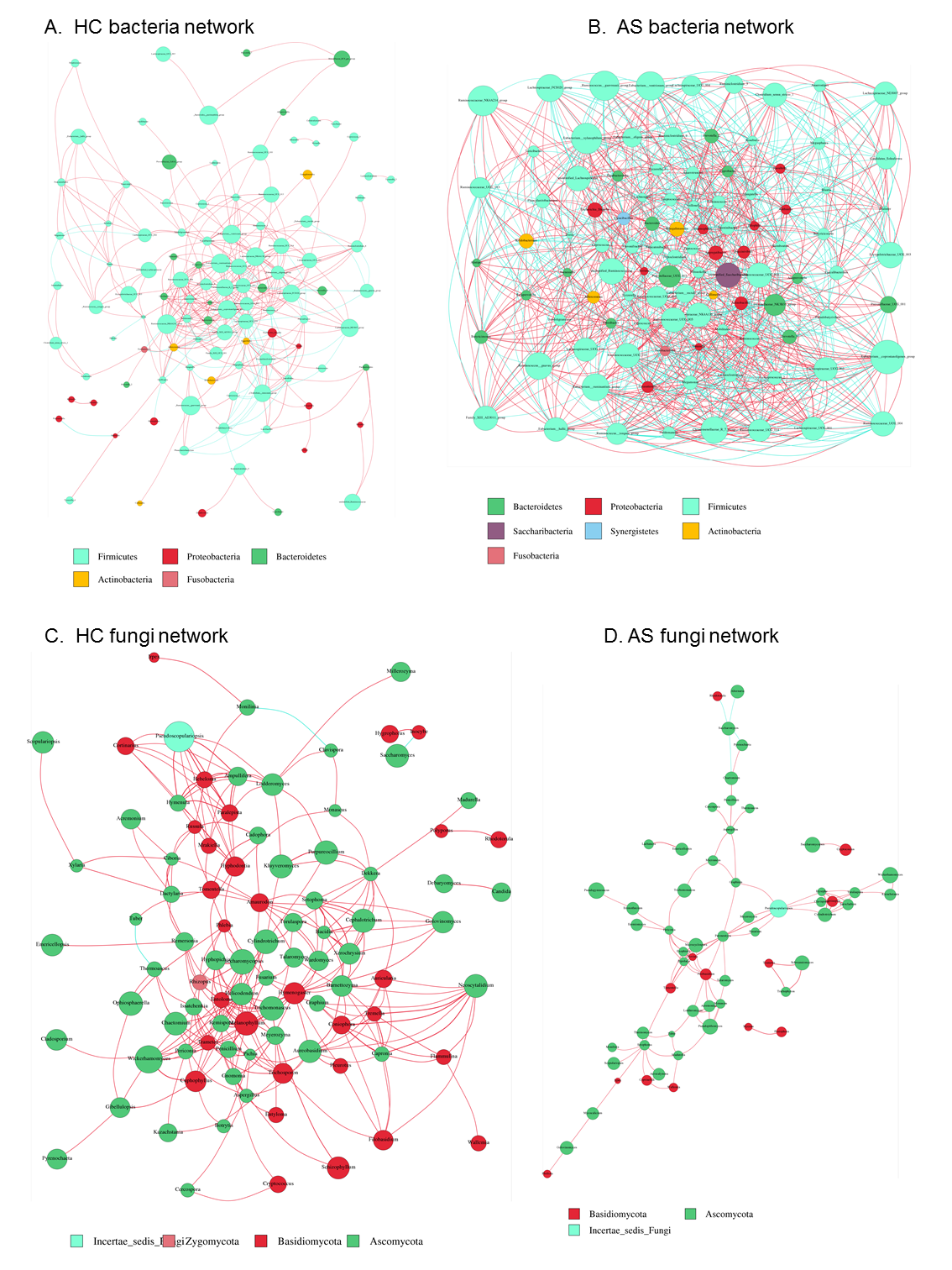

Supplement: FIG S6 [file mSystems.00176-18-sf006.tif]

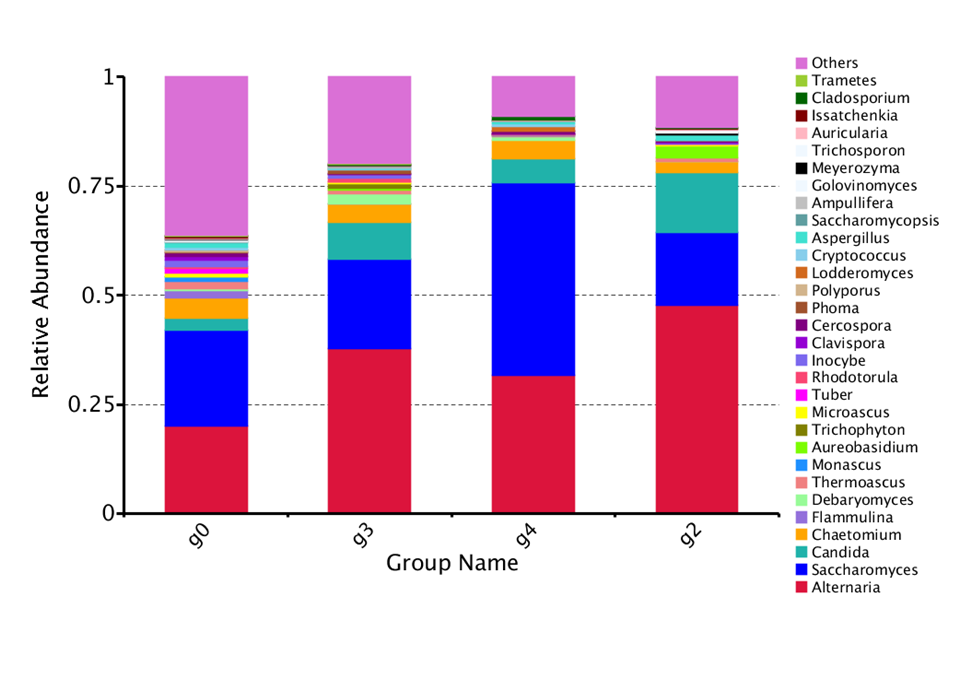

Supplement: FIG S7 [file mSystems.00176-18-sf007.tif]
