# Supplementary material for: Altered Bacterial-Fungal Interkingdom Networks in the Guts of Ankylosing Spondylitis Patients
Source: mSystems. 2019 Mar 26;4(2):e00176-18. doi: 10.1128/mSystems.00176-18 (PMC6435815; doi:10.1128/mSystems.00176-18)
Supplement: TABLE S1 [file mSystems.00176-18-st001.docx]

**Table S1 The statistic test of the observed species between different groups**

| Groups | Difference | P value | UCL | LCL |
| --- | --- | --- | --- | --- |
| BL - No | -10.3437500 | 0.0339 * | -19.854962 | -0.8325382 |
| BL - NS | -5.4236111 | 0.3090 | -16.096781 | 5.2495589 |
| BL - TN | -5.1125000 | 0.4125 | -17.634592 | 7.4095922 |
| No - NS | 4.9201389 | 0.2823 | -4.232029 | 14.0723067 |
| No - TN | 5.2312500 | 0.3515 | -6.022568 | 16.4850676 |
| NS - TN | 0.3111111 | 0.9591 | -11.940494 | 12.5627159 |
