# Supplementary material for: Altered Bacterial-Fungal Interkingdom Networks in the Guts of Ankylosing Spondylitis Patients
Source: mSystems. 2019 Mar 26;4(2):e00176-18. doi: 10.1128/mSystems.00176-18 (PMC6435815; doi:10.1128/mSystems.00176-18)
Supplement: TABLE S2 [file mSystems.00176-18-st002.docx]

**Table S2 The statistic test of the shannon index between different groups**

| Groups | Difference | P value | UCL | LCL |
| --- | --- | --- | --- | --- |
| BL - No | -14.062500 | 0.0021 ** | -22.667221 | -5.457779 |
| BL - NS | -11.152778 | 0.0249 * | -20.808714 | -1.496842 |
| BL - TN | -2.375000 | 0.6728 | -13.703642 | 8.953642 |
| No - NS | 2.909722 | 0.4800 | -5.370175 | 11.189619 |
| No - TN | 11.687500 | 0.0257 * | 1.506256 | 21.868744 |
| NS - TN | 8.777778 | 0.1168 | -2.306156 | 19.861712 |
