# Supplementary material for: Altered Bacterial-Fungal Interkingdom Networks in the Guts of Ankylosing Spondylitis Patients
Source: mSystems. 2019 Mar 26;4(2):e00176-18. doi: 10.1128/mSystems.00176-18 (PMC6435815; doi:10.1128/mSystems.00176-18)
Supplement: TABLE S3 [file mSystems.00176-18-st003.docx]

**Table S3 The statistic test of the Simpson index between different groups**

| Groups | Difference | P value | UCL | LCL |
| --- | --- | --- | --- | --- |
| BL - No | -13.718750 | 0.0025 ** | -22.2537588 | -5.183741 |
| BL - NS | -12.555556 | 0.0117 * | -22.1332626 | -2.977849 |
| BL - TN | -1.900000 | 0.7332 | -13.1368612 | 9.336861 |
| No - NS | 1.163194 | 0.7752 | -7.0496216 | 9.376010 |
| No - TN | 11.818750 | 0.0232 * | 1.7199914 | 21.917509 |
| NS - TN | 10.655556 | 0.0571 | -0.3385803 | 21.649691 |
